# Tetra­ethyl 1,1′-(ethane-1,2-di­yl)bis­(2,5-dimethyl-1*H*-pyrrole-3,4-dicarboxyl­ate)

**DOI:** 10.1107/S1600536810044119

**Published:** 2010-11-06

**Authors:** Shi-Fan Wang, Chao Li, Shuai Chen

**Affiliations:** a School of Ocean, Hainan University, Haikou 570228, People’s Republic of China; b Experimental Teaching Center of Marine Biology, Hainan University, Haikou 570228, People’s Republic of China

## Abstract

The asymmetric unit of the title compound, C_26_H_36_N_2_O_8_, comprises two independent mol­ecules. In each mol­ecule, the two pyrrole rings are linked by a –CH_2_CH_2_– bridge, with dihedral angles between the two pyrrole rings of 14.5 (3) and 16.4 (3)° in the two mol­ecules. Each pyrrole ring carries 2- and 5-methyl substituents and eth­oxy­carbonyl groups at the 3- and 5-positions.

## Related literature

For background to the biological applications of bis­pyrrole and its derivatives, see: Dairi *et al.* (2006 ▶); Bordner & Rapoport (1965 ▶); Rapoport & Castagnoli (1962 ▶). For the synthesis and biological properties of pyrrole derivatives containing *N*-substituent groups, see: Banik *et al.* (2004 ▶); Sagyam *et al.* (2007 ▶). For details of the the Paal–Knorr condensation reaction, see Amarnath *et al.* (1991 ▶). For representative bond-length data, see: Allen *et al.* (1987 ▶).
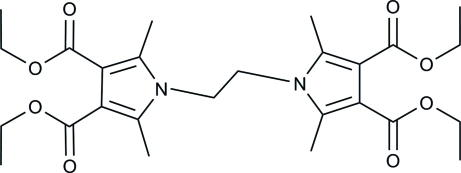


## Experimental

### 

#### Crystal data

C_26_H_36_N_2_O_8_
*M* *_r_* = 504.57
Monoclinic, 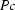

*a* = 12.891 (3) Å
*b* = 13.743 (3) Å
*c* = 16.717 (3) Å
β = 113.350 (14)°
*V* = 2719.0 (10) Å^3^
*Z* = 4
Mo *K*α radiation
μ = 0.09 mm^−1^
*T* = 298 K
0.20 × 0.18 × 0.17 mm

#### Data collection

Bruker SMART 1000 CCD diffractometer
Absorption correction: multi-scan (*SADABS*; Bruker, 2001 ▶) *T*_min_ = 0.982, *T*_max_ = 0.985
5726 measured reflections
5726 independent reflections
3020 reflections with *I* > 2σ(*I*)
*R*_int_ = 0.000

#### Refinement

*R*[*F* ^2^ > 2σ(*F* ^2^)] = 0.062
*wR*(*F* ^2^) = 0.194
*S* = 0.99
5726 reflections
663 parameters
2 restraints
H-atom parameters constrained
Δρ_max_ = 0.29 e Å^−3^
Δρ_min_ = −0.18 e Å^−3^

### 

Data collection: *SMART* (Bruker, 2007 ▶); cell refinement: *SAINT* (Bruker, 2007 ▶); data reduction: *SAINT*; program(s) used to solve structure: *SHELXTL* (Sheldrick, 2008 ▶); program(s) used to refine structure: *SHELXTL*; molecular graphics: *SHELXTL*; software used to prepare material for publication: *SHELXTL*.

## Supplementary Material

Crystal structure: contains datablocks global, I. DOI: 10.1107/S1600536810044119/sj5043sup1.cif

Structure factors: contains datablocks I. DOI: 10.1107/S1600536810044119/sj5043Isup2.hkl

Additional supplementary materials:  crystallographic information; 3D view; checkCIF report

## Figures and Tables

**Fig. 1.**
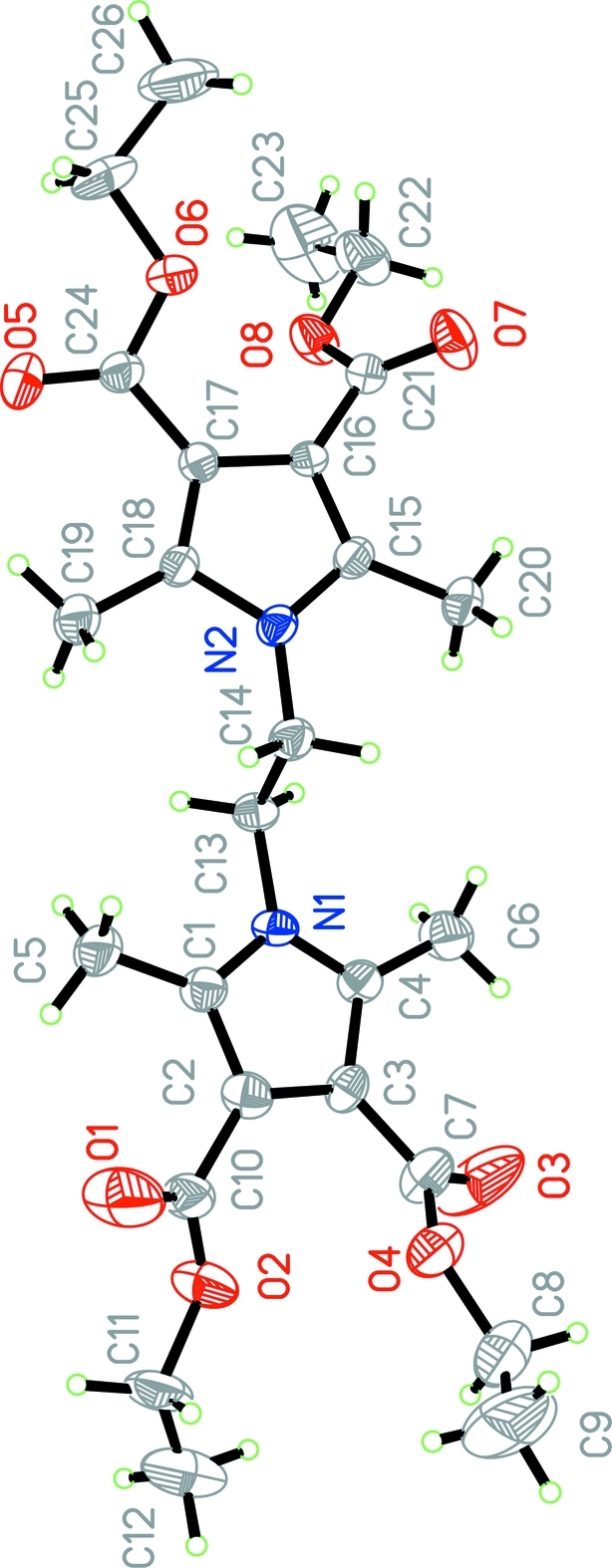
The structure of molecule A of the title compound with atom labels and 30% probability displacement ellipsoids for non-H atoms.

**Fig. 2.**
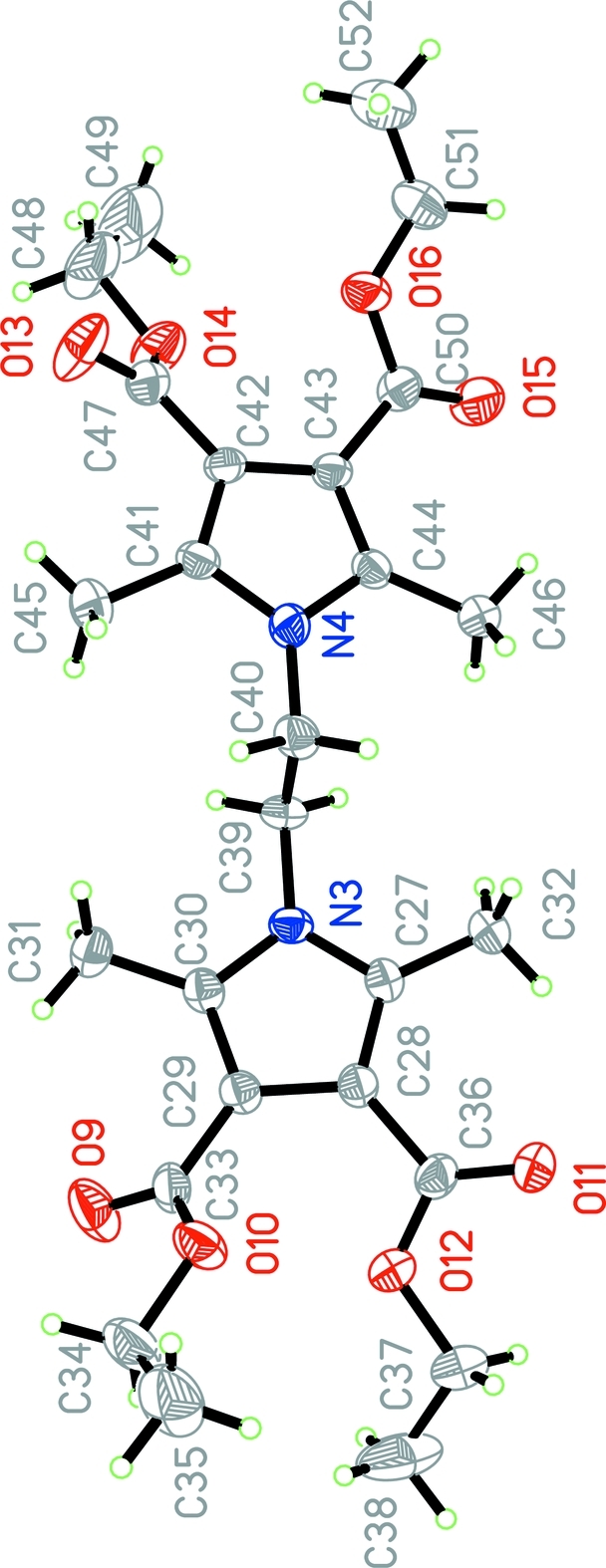
The structure of molecule B of the title compound with atom labels and 30% probability displacement ellipsoids for non-H atoms.

## supplementary crystallographic information

### Comment

Bis-pyrrole and its derivatives play important roles in some bioactive pyrrole natural products, (Dairi *et al.*, 2006; Bordner & Rapoport, 1965; Rapoport & Castagnoli, 1962). Recently, the synthesis of pyrrole derivatives with N-substituent groups aroused great interest because of their significant biological activity (Banik *et al.*, 2004; Sagyam *et al.*, 2007). As an intermediate for further synthesis of pyrrole derivatives containing N-substituent groups, we have prepared the title compound by the Paal-Knorr condensation reaction (Amarnath *et al.*, 1991) and obtained its strcuture is reported here.

In the asymmetric unit of the title compound, Fig. 1, there are two independent molecules, A and B. The dihedral angle between the two pyrrole rings in one molecule is 14.5 (3)°, and that in the other molecule is 16.4 (3)°. All the bond lengths are within normal ranges (Allen *et al.*, 1987).

### Experimental

12.8 ml of ethyl acetoacetate (0.10 mol) and 2.28 g (0.10 mol) of sodium metal were added into 300 ml dry ether under stirring at room temperature. Then, the mixed solution was refluxed for 24 h till the Na was depleted. 100 ml dry ether solution contained 12.5 g (0.050 mol) I_2_ was added dropwise. After all of the iodine solution had been added, the reaction mixture was refluxed for 12 h and then cooled to room temperature. The undissolved solid was filtered and the ether solution was evaporated to yield diacetyl butanedioic acid diethyl ester as a gray solid (8.64 g, 67%). 5.20 g (20 mmol) of diacetyl butanedioic acid diethyl ester and 0.60 g (10 mmol) of ethylenediamine were dissolved into the mixed solution of ethanol and acetic acid (*v*/*v*, 5:1). The mixture was then refluxed for 6 h and evaporated to remove the ethanol. The residue was poured into water to give the title compound as a white solid (4.91 g, 97%). A little of the solid was dissolved in mixed solvent of acetone-water (*v*/*v*, 20:1). After standing in air over a period of about five days, the acetone is evaporated, colourless crystals suitable for X-ray diffraction analysis were formed at the bottom of the vessel.

### Refinement

H atoms were positioned geometrically and refined using the riding-model approximation, with C–H = 0.93–0.97 Å, and *U*_iso_(H) = 1.2*U*_eq_(C) or *U*_iso_(H) = 1.5*U*_eq_(methyl C). In the absence of significant anomalous dispersion effects, Friedel pairs were averaged.  Displacement parameters on some of the atoms particularly of the ethyl groups of the ethylcarboxylate substituents were unusually large. However, a suitable disorder model could not be found for them.

### Crystal data

**Table:**

C_26_H_36_N_2_O_8_	*F*(000) = 1080
*M**_r_* = 504.57	*D*_x_ = 1.233 Mg m^−^^3^
Monoclinic, *P**c*	Mo *K*α radiation, λ = 0.71073 Å
Hall symbol: P -2yc	Cell parameters from 2429 reflections
*a* = 12.891 (3) Å	θ = 2.5–24.5°
*b* = 13.743 (3) Å	µ = 0.09 mm^−^^1^
*c* = 16.717 (3) Å	*T* = 298 K
β = 113.350 (14)°	Block, colourless
*V* = 2719.0 (10) Å^3^	0.20 × 0.18 × 0.17 mm
*Z* = 4	

### Data collection

**Table:**

Bruker SMART 1000 CCD diffractometer	5726 independent reflections
Radiation source: fine-focus sealed tube	3020 reflections with *I* > 2σ(*I*)
graphite	*R*_int_ = 0.0000
ω scans	θ_max_ = 27.0°, θ_min_ = 1.5°
Absorption correction: multi-scan (*SADABS*; Bruker, 2001)	*h* = −16→15
*T*_min_ = 0.982, *T*_max_ = 0.985	*k* = 0→17
5726 measured reflections	*l* = 0→21

### Refinement

**Table:**

Refinement on *F*^2^	Primary atom site location: structure-invariant direct methods
Least-squares matrix: full	Secondary atom site location: difference Fourier map
*R*[*F*^2^ > 2σ(*F*^2^)] = 0.062	Hydrogen site location: inferred from neighbouring sites
*wR*(*F*^2^) = 0.194	H-atom parameters constrained
*S* = 0.99	*w* = 1/[σ^2^(*F*_o_^2^) + (0.1027*P*)^2^] where *P* = (*F*_o_^2^ + 2*F*_c_^2^)/3
5726 reflections	(Δ/σ)_max_ = 0.001
663 parameters	Δρ_max_ = 0.29 e Å^−^^3^
2 restraints	Δρ_min_ = −0.18 e Å^−^^3^

### Special details

**Table:**

Geometry. All e.s.d.'s (except the e.s.d. in the dihedral angle between two l.s. planes) are estimated using the full covariance matrix. The cell e.s.d.'s are taken into account individually in the estimation of e.s.d.'s in distances, angles and torsion angles; correlations between e.s.d.'s in cell parameters are only used when they are defined by crystal symmetry. An approximate (isotropic) treatment of cell e.s.d.'s is used for estimating e.s.d.'s involving l.s. planes.
Refinement. Refinement of *F*^2^ against ALL reflections. The weighted *R*-factor *wR* and goodness of fit *S* are based on *F*^2^, conventional *R*-factors *R* are based on *F*, with *F* set to zero for negative *F*^2^. The threshold expression of *F*^2^ > σ(*F*^2^) is used only for calculating *R*-factors(gt) *etc*. and is not relevant to the choice of reflections for refinement. *R*-factors based on *F*^2^ are statistically about twice as large as those based on *F*, and *R*- factors based on ALL data will be even larger.

### Fractional atomic coordinates and isotropic or equivalent isotropic displacement parameters (Å^2^)

**Table:**

	*x*	*y*	*z*	*U*_iso_*/*U*_eq_	
O1	0.6385 (7)	0.3819 (5)	0.1367 (4)	0.133 (3)	
O2	0.6668 (5)	0.4974 (4)	0.2316 (3)	0.0819 (15)	
O3	0.7288 (7)	0.4428 (7)	0.4836 (4)	0.173 (5)	
O4	0.8112 (5)	0.4230 (4)	0.3945 (3)	0.0933 (16)	
O5	0.0805 (4)	−0.1379 (4)	0.0950 (3)	0.0823 (15)	
O6	0.1269 (4)	−0.2452 (3)	0.2036 (3)	0.0707 (13)	
O7	0.3049 (4)	−0.2263 (4)	0.4369 (3)	0.0765 (14)	
O8	0.1351 (3)	−0.1624 (3)	0.3687 (3)	0.0678 (12)	
N1	0.4959 (5)	0.2503 (4)	0.3016 (3)	0.0456 (13)	
N2	0.3699 (5)	0.0020 (4)	0.2865 (3)	0.0449 (13)	
C1	0.5129 (6)	0.2823 (5)	0.2310 (4)	0.0522 (17)	
C2	0.5991 (6)	0.3500 (5)	0.2616 (4)	0.0565 (19)	
C3	0.6301 (6)	0.3564 (5)	0.3544 (4)	0.0519 (17)	
C4	0.5637 (5)	0.2912 (5)	0.3764 (4)	0.0505 (17)	
C5	0.4492 (7)	0.2512 (6)	0.1411 (4)	0.075 (2)	
H5A	0.4740	0.1876	0.1326	0.112*	
H5B	0.4618	0.2964	0.1020	0.112*	
H5C	0.3701	0.2493	0.1295	0.112*	
C6	0.5673 (7)	0.2669 (6)	0.4622 (5)	0.078 (2)	
H6A	0.6291	0.3005	0.5060	0.117*	
H6B	0.5774	0.1980	0.4714	0.117*	
H6C	0.4977	0.2861	0.4658	0.117*	
C7	0.7231 (7)	0.4142 (7)	0.4190 (6)	0.077 (3)	
C8	0.9070 (9)	0.4896 (9)	0.4463 (6)	0.130 (4)	
H8A	0.8798	0.5560	0.4420	0.156*	
H8B	0.9374	0.4707	0.5072	0.156*	
C9	0.9903 (9)	0.4839 (11)	0.4143 (11)	0.196 (7)	
H9A	1.0497	0.5290	0.4451	0.294*	
H9B	0.9590	0.4998	0.3534	0.294*	
H9C	1.0203	0.4191	0.4221	0.294*	
C10	0.6377 (7)	0.4101 (6)	0.2051 (5)	0.065 (2)	
C11	0.7157 (8)	0.5562 (7)	0.1838 (5)	0.103 (3)	
H11A	0.7847	0.5262	0.1856	0.124*	
H11B	0.6633	0.5614	0.1234	0.124*	
C12	0.7393 (9)	0.6497 (7)	0.2222 (8)	0.126 (4)	
H12A	0.6773	0.6926	0.1919	0.189*	
H12B	0.8068	0.6747	0.2185	0.189*	
H12C	0.7499	0.6454	0.2823	0.189*	
C13	0.4084 (5)	0.1770 (4)	0.2939 (4)	0.0460 (16)	
H13A	0.3417	0.1892	0.2414	0.055*	
H13B	0.3874	0.1826	0.3434	0.055*	
C14	0.4509 (5)	0.0752 (5)	0.2907 (4)	0.0518 (18)	
H14A	0.4695	0.0694	0.2400	0.062*	
H14B	0.5196	0.0644	0.3420	0.062*	
C15	0.3637 (5)	−0.0487 (5)	0.3554 (4)	0.0467 (16)	
C16	0.2756 (5)	−0.1119 (4)	0.3244 (3)	0.0388 (14)	
C17	0.2231 (5)	−0.0999 (5)	0.2330 (4)	0.0431 (16)	
C18	0.2818 (5)	−0.0299 (5)	0.2104 (4)	0.0443 (16)	
C19	0.2710 (7)	0.0107 (6)	0.1232 (4)	0.071 (2)	
H19A	0.2518	0.0785	0.1201	0.106*	
H19B	0.2127	−0.0238	0.0772	0.106*	
H19C	0.3414	0.0031	0.1170	0.106*	
C20	0.4437 (6)	−0.0241 (6)	0.4472 (4)	0.063 (2)	
H20A	0.4261	−0.0633	0.4877	0.094*	
H20B	0.4360	0.0434	0.4585	0.094*	
H20C	0.5200	−0.0368	0.4539	0.094*	
C21	0.2419 (6)	−0.1726 (5)	0.3819 (4)	0.0485 (16)	
C22	0.0935 (7)	−0.2215 (6)	0.4220 (6)	0.089 (2)	
H22A	0.0929	−0.2895	0.4063	0.107*	
H22B	0.1432	−0.2144	0.4830	0.107*	
C23	−0.0193 (8)	−0.1911 (9)	0.4089 (8)	0.152 (5)	
H23A	−0.0162	−0.1285	0.4352	0.228*	
H23B	−0.0517	−0.2376	0.4351	0.228*	
H23C	−0.0649	−0.1870	0.3476	0.228*	
C24	0.1352 (6)	−0.1593 (5)	0.1703 (4)	0.0509 (18)	
C25	0.0423 (7)	−0.3109 (6)	0.1489 (6)	0.098 (3)	
H25A	0.0602	−0.3318	0.1005	0.117*	
H25B	−0.0310	−0.2794	0.1259	0.117*	
C26	0.0408 (9)	−0.3958 (7)	0.2035 (7)	0.127 (4)	
H26A	0.1167	−0.4177	0.2358	0.191*	
H26B	−0.0026	−0.4474	0.1668	0.191*	
H26C	0.0072	−0.3770	0.2432	0.191*	
O9	0.5561 (4)	0.9699 (4)	0.1424 (3)	0.0949 (18)	
O10	0.7295 (4)	0.9161 (3)	0.2169 (3)	0.0663 (12)	
O11	0.7871 (4)	0.8930 (4)	0.4889 (3)	0.0920 (17)	
O12	0.7420 (4)	0.9967 (3)	0.3800 (3)	0.0659 (12)	
O13	0.1588 (5)	0.2854 (4)	0.1302 (3)	0.0999 (18)	
O14	0.0572 (4)	0.3395 (4)	0.2025 (3)	0.0784 (14)	
O15	0.2457 (5)	0.3679 (4)	0.4697 (3)	0.0921 (17)	
O16	0.2027 (4)	0.2573 (3)	0.3677 (3)	0.0643 (12)	
N3	0.4994 (4)	0.7494 (4)	0.3027 (3)	0.0445 (13)	
N4	0.3782 (5)	0.5013 (4)	0.2989 (3)	0.0490 (14)	
C27	0.5838 (5)	0.7816 (5)	0.3766 (4)	0.0465 (17)	
C28	0.6412 (5)	0.8515 (5)	0.3522 (4)	0.0469 (17)	
C29	0.5865 (5)	0.8617 (5)	0.2585 (4)	0.0467 (15)	
C30	0.4989 (5)	0.7976 (5)	0.2309 (4)	0.0466 (15)	
C31	0.4202 (6)	0.7749 (5)	0.1407 (4)	0.0597 (19)	
H31A	0.4222	0.7064	0.1303	0.090*	
H31B	0.4425	0.8102	0.1005	0.090*	
H31C	0.3450	0.7935	0.1326	0.090*	
C32	0.6010 (6)	0.7457 (5)	0.4635 (4)	0.063 (2)	
H32A	0.6363	0.6829	0.4725	0.095*	
H32B	0.5294	0.7406	0.4683	0.095*	
H32C	0.6487	0.7902	0.5068	0.095*	
C33	0.6200 (6)	0.9234 (6)	0.2012 (4)	0.0560 (18)	
C34	0.7673 (6)	0.9755 (6)	0.1622 (5)	0.086 (2)	
H34A	0.7564	1.0438	0.1710	0.103*	
H34B	0.7245	0.9599	0.1013	0.103*	
C35	0.8860 (7)	0.9555 (8)	0.1858 (6)	0.117 (3)	
H35A	0.9284	0.9768	0.2445	0.176*	
H35B	0.9116	0.9895	0.1469	0.176*	
H35C	0.8967	0.8868	0.1817	0.176*	
C36	0.7301 (5)	0.9140 (6)	0.4137 (4)	0.0539 (19)	
C37	0.8286 (7)	1.0645 (6)	0.4332 (5)	0.097 (3)	
H37A	0.8177	1.0789	0.4861	0.116*	
H37B	0.9025	1.0349	0.4495	0.116*	
C38	0.8238 (8)	1.1506 (6)	0.3875 (8)	0.129 (4)	
H38A	0.8778	1.1961	0.4248	0.193*	
H38B	0.7493	1.1778	0.3685	0.193*	
H38C	0.8410	1.1370	0.3377	0.193*	
C39	0.4157 (5)	0.6754 (4)	0.3000 (4)	0.0459 (16)	
H39A	0.3479	0.6841	0.2474	0.055*	
H39B	0.3955	0.6843	0.3495	0.055*	
C40	0.4601 (6)	0.5742 (5)	0.3016 (4)	0.0532 (19)	
H40A	0.4803	0.5655	0.2520	0.064*	
H40B	0.5278	0.5656	0.3541	0.064*	
C41	0.3053 (6)	0.4518 (5)	0.2249 (4)	0.0477 (16)	
C42	0.2423 (5)	0.3905 (5)	0.2500 (4)	0.0458 (16)	
C43	0.2723 (5)	0.4021 (4)	0.3397 (4)	0.0422 (15)	
C44	0.3576 (5)	0.4694 (4)	0.3699 (3)	0.0446 (16)	
C45	0.3057 (7)	0.4778 (6)	0.1375 (4)	0.068 (2)	
H45A	0.2878	0.5455	0.1258	0.103*	
H45B	0.2504	0.4392	0.0930	0.103*	
H45C	0.3791	0.4652	0.1378	0.103*	
C46	0.4253 (6)	0.5073 (5)	0.4589 (4)	0.0613 (19)	
H46A	0.4093	0.4697	0.5010	0.092*	
H46B	0.4060	0.5742	0.4624	0.092*	
H46C	0.5043	0.5026	0.4705	0.092*	
C47	0.1503 (7)	0.3337 (6)	0.1879 (4)	0.062 (2)	
C48	−0.0347 (8)	0.2802 (9)	0.1476 (6)	0.123 (4)	
H48A	−0.0690	0.3093	0.0902	0.148*	
H48B	−0.0069	0.2162	0.1415	0.148*	
C49	−0.1135 (10)	0.2716 (11)	0.1827 (9)	0.194 (7)	
H49A	−0.0957	0.3158	0.2308	0.291*	
H49B	−0.1135	0.2062	0.2028	0.291*	
H49C	−0.1869	0.2869	0.1392	0.291*	
C50	0.2382 (6)	0.3448 (5)	0.3992 (4)	0.0548 (18)	
C51	0.1578 (7)	0.1949 (6)	0.4156 (5)	0.089 (2)	
H51A	0.2116	0.1905	0.4757	0.106*	
H51B	0.0886	0.2231	0.4154	0.106*	
C52	0.1351 (8)	0.1011 (6)	0.3792 (6)	0.099 (3)	
H52A	0.0690	0.1030	0.3257	0.148*	
H52B	0.1224	0.0575	0.4192	0.148*	
H52C	0.1984	0.0787	0.3677	0.148*	

### Atomic displacement parameters (Å^2^)

**Table:**

	*U*^11^	*U*^22^	*U*^33^	*U*^12^	*U*^13^	*U*^23^
O1	0.213 (8)	0.129 (5)	0.093 (4)	−0.075 (5)	0.100 (5)	−0.036 (4)
O2	0.117 (4)	0.063 (3)	0.089 (3)	−0.018 (3)	0.067 (3)	−0.002 (3)
O3	0.164 (7)	0.287 (11)	0.093 (4)	−0.142 (7)	0.079 (5)	−0.110 (6)
O4	0.076 (3)	0.111 (4)	0.080 (3)	−0.031 (3)	0.016 (3)	−0.005 (3)
O5	0.075 (3)	0.097 (4)	0.052 (3)	−0.018 (3)	0.001 (2)	0.001 (2)
O6	0.069 (3)	0.050 (3)	0.075 (3)	−0.019 (2)	0.009 (2)	0.002 (2)
O7	0.071 (3)	0.097 (4)	0.066 (3)	0.014 (3)	0.031 (2)	0.035 (3)
O8	0.050 (3)	0.079 (3)	0.082 (3)	0.005 (2)	0.034 (2)	0.031 (2)
N1	0.055 (3)	0.034 (3)	0.052 (3)	−0.007 (3)	0.026 (3)	−0.003 (2)
N2	0.048 (3)	0.049 (3)	0.036 (3)	−0.010 (3)	0.015 (2)	−0.006 (2)
C1	0.058 (4)	0.052 (4)	0.050 (4)	−0.006 (4)	0.025 (3)	−0.006 (3)
C2	0.063 (4)	0.061 (5)	0.058 (4)	−0.002 (4)	0.037 (3)	−0.001 (3)
C3	0.051 (4)	0.061 (4)	0.042 (3)	−0.002 (4)	0.017 (3)	−0.014 (3)
C4	0.048 (4)	0.061 (4)	0.049 (4)	−0.006 (3)	0.026 (3)	−0.003 (3)
C5	0.086 (5)	0.086 (5)	0.046 (4)	−0.027 (4)	0.020 (3)	−0.009 (4)
C6	0.101 (6)	0.083 (5)	0.058 (4)	−0.022 (5)	0.039 (4)	−0.006 (4)
C7	0.059 (5)	0.106 (8)	0.066 (5)	−0.016 (5)	0.024 (4)	−0.019 (5)
C8	0.089 (7)	0.171 (11)	0.097 (6)	−0.036 (7)	0.004 (5)	0.006 (7)
C9	0.076 (7)	0.241 (17)	0.268 (18)	−0.045 (8)	0.064 (10)	0.011 (13)
C10	0.076 (5)	0.072 (6)	0.054 (4)	−0.020 (4)	0.034 (4)	−0.003 (4)
C11	0.143 (7)	0.101 (7)	0.085 (5)	−0.062 (6)	0.065 (5)	0.003 (5)
C12	0.132 (8)	0.086 (7)	0.193 (11)	−0.014 (6)	0.099 (8)	0.028 (7)
C13	0.051 (4)	0.034 (4)	0.055 (4)	−0.001 (3)	0.024 (3)	−0.007 (3)
C14	0.048 (4)	0.061 (5)	0.048 (4)	−0.012 (4)	0.021 (3)	−0.002 (3)
C15	0.041 (3)	0.048 (4)	0.046 (3)	−0.002 (3)	0.012 (3)	−0.004 (3)
C16	0.045 (3)	0.030 (3)	0.038 (3)	−0.003 (3)	0.012 (3)	−0.003 (3)
C17	0.041 (3)	0.040 (4)	0.045 (3)	0.000 (3)	0.014 (3)	0.001 (3)
C18	0.041 (3)	0.049 (4)	0.038 (3)	−0.004 (3)	0.011 (3)	−0.003 (3)
C19	0.084 (5)	0.083 (5)	0.041 (3)	−0.024 (4)	0.021 (3)	−0.004 (3)
C20	0.064 (4)	0.074 (5)	0.040 (3)	−0.008 (4)	0.009 (3)	−0.003 (3)
C21	0.050 (4)	0.044 (4)	0.047 (3)	−0.003 (3)	0.014 (3)	0.002 (3)
C22	0.094 (6)	0.097 (6)	0.094 (5)	−0.003 (5)	0.057 (5)	0.030 (4)
C23	0.097 (7)	0.218 (13)	0.183 (10)	0.016 (7)	0.100 (7)	0.072 (9)
C24	0.050 (4)	0.053 (5)	0.049 (4)	−0.012 (3)	0.018 (3)	−0.001 (3)
C25	0.084 (6)	0.083 (6)	0.109 (6)	−0.050 (5)	0.020 (5)	−0.027 (5)
C26	0.138 (9)	0.084 (7)	0.156 (9)	−0.064 (6)	0.052 (7)	−0.029 (6)
O9	0.062 (3)	0.142 (5)	0.073 (3)	0.011 (3)	0.019 (3)	0.056 (3)
O10	0.057 (3)	0.079 (3)	0.070 (3)	−0.001 (2)	0.034 (2)	0.023 (2)
O11	0.085 (4)	0.114 (4)	0.051 (3)	−0.044 (3)	−0.001 (3)	0.011 (3)
O12	0.061 (3)	0.070 (3)	0.058 (2)	−0.023 (2)	0.014 (2)	−0.003 (2)
O13	0.095 (4)	0.127 (4)	0.073 (3)	−0.027 (3)	0.029 (3)	−0.055 (3)
O14	0.049 (3)	0.102 (4)	0.083 (3)	−0.020 (2)	0.025 (2)	−0.025 (3)
O15	0.137 (5)	0.094 (4)	0.065 (3)	−0.050 (3)	0.062 (3)	−0.025 (3)
O16	0.081 (3)	0.057 (3)	0.063 (3)	−0.019 (2)	0.037 (2)	0.000 (2)
N3	0.039 (3)	0.042 (3)	0.053 (3)	−0.005 (2)	0.019 (2)	0.004 (2)
N4	0.053 (3)	0.057 (4)	0.041 (3)	−0.006 (3)	0.023 (2)	−0.003 (2)
C27	0.049 (4)	0.051 (4)	0.040 (3)	0.002 (3)	0.018 (3)	0.003 (3)
C28	0.037 (3)	0.061 (4)	0.042 (3)	−0.002 (3)	0.015 (3)	0.004 (3)
C29	0.038 (3)	0.057 (4)	0.047 (3)	0.001 (3)	0.020 (3)	0.006 (3)
C30	0.041 (3)	0.058 (4)	0.045 (3)	0.003 (3)	0.021 (3)	0.004 (3)
C31	0.054 (4)	0.069 (5)	0.047 (3)	−0.002 (3)	0.010 (3)	−0.004 (3)
C32	0.070 (4)	0.068 (5)	0.047 (3)	−0.012 (4)	0.018 (3)	0.011 (3)
C33	0.049 (4)	0.078 (5)	0.037 (3)	−0.002 (4)	0.013 (3)	0.006 (3)
C34	0.078 (5)	0.111 (7)	0.079 (5)	−0.011 (4)	0.044 (4)	0.028 (4)
C35	0.079 (6)	0.188 (10)	0.098 (6)	−0.007 (6)	0.049 (5)	0.032 (6)
C36	0.037 (4)	0.070 (6)	0.050 (4)	−0.007 (3)	0.012 (3)	−0.001 (3)
C37	0.106 (6)	0.081 (6)	0.088 (5)	−0.046 (5)	0.023 (5)	−0.008 (4)
C38	0.086 (6)	0.058 (5)	0.216 (11)	−0.019 (5)	0.031 (7)	0.005 (6)
C39	0.045 (4)	0.032 (4)	0.064 (4)	−0.002 (3)	0.025 (3)	0.003 (3)
C40	0.046 (4)	0.065 (5)	0.054 (4)	−0.004 (4)	0.026 (3)	0.006 (3)
C41	0.060 (4)	0.040 (4)	0.049 (3)	−0.006 (3)	0.029 (3)	0.001 (3)
C42	0.056 (4)	0.038 (3)	0.048 (3)	−0.011 (3)	0.025 (3)	−0.005 (3)
C43	0.046 (4)	0.040 (4)	0.044 (3)	−0.007 (3)	0.021 (3)	−0.002 (3)
C44	0.053 (4)	0.043 (4)	0.042 (3)	−0.001 (3)	0.023 (3)	0.004 (3)
C45	0.083 (5)	0.081 (5)	0.046 (4)	−0.004 (4)	0.032 (3)	0.003 (3)
C46	0.069 (4)	0.060 (4)	0.049 (3)	−0.015 (3)	0.017 (3)	0.002 (3)
C47	0.076 (5)	0.063 (5)	0.050 (4)	−0.015 (4)	0.028 (4)	−0.005 (3)
C48	0.081 (6)	0.192 (11)	0.090 (6)	−0.079 (7)	0.025 (5)	−0.036 (6)
C49	0.118 (10)	0.270 (18)	0.150 (11)	−0.095 (11)	0.007 (9)	−0.029 (11)
C50	0.059 (4)	0.062 (5)	0.052 (4)	−0.010 (3)	0.031 (3)	−0.010 (3)
C51	0.107 (6)	0.087 (6)	0.082 (5)	−0.023 (5)	0.047 (4)	0.018 (5)
C52	0.123 (7)	0.070 (6)	0.116 (7)	−0.006 (5)	0.061 (6)	0.010 (5)

### Geometric parameters (Å, °)

**Table:**

O1—C10	1.210 (9)	O9—C33	1.187 (8)
O2—C10	1.282 (9)	O10—C33	1.333 (8)
O2—C11	1.444 (8)	O10—C34	1.446 (7)
O3—C7	1.123 (9)	O11—C36	1.212 (8)
O4—C7	1.356 (10)	O12—C36	1.305 (8)
O4—C8	1.505 (11)	O12—C37	1.454 (8)
O5—C24	1.210 (7)	O13—C47	1.211 (9)
O6—C24	1.327 (8)	O14—C47	1.318 (9)
O6—C25	1.432 (7)	O14—C48	1.431 (8)
O7—C21	1.208 (7)	O15—C50	1.186 (8)
O8—C21	1.314 (8)	O16—C50	1.321 (8)
O8—C22	1.456 (7)	O16—C51	1.443 (8)
N1—C4	1.332 (8)	N3—C27	1.356 (7)
N1—C1	1.357 (9)	N3—C30	1.368 (8)
N1—C13	1.480 (8)	N3—C39	1.470 (8)
N2—C15	1.375 (8)	N4—C44	1.386 (8)
N2—C18	1.399 (7)	N4—C41	1.397 (8)
N2—C14	1.432 (8)	N4—C40	1.443 (9)
C1—C2	1.384 (10)	C27—C28	1.369 (8)
C1—C5	1.462 (9)	C27—C32	1.466 (9)
C2—C3	1.443 (9)	C28—C29	1.448 (8)
C2—C10	1.482 (10)	C28—C36	1.475 (9)
C3—C4	1.386 (9)	C29—C30	1.361 (9)
C3—C7	1.486 (10)	C29—C33	1.467 (10)
C4—C6	1.456 (9)	C30—C31	1.479 (8)
C5—H5A	0.9600	C31—H31A	0.9600
C5—H5B	0.9600	C31—H31B	0.9600
C5—H5C	0.9600	C31—H31C	0.9600
C6—H6A	0.9600	C32—H32A	0.9600
C6—H6B	0.9600	C32—H32B	0.9600
C6—H6C	0.9600	C32—H32C	0.9600
C8—C9	1.378 (15)	C34—C35	1.447 (11)
C8—H8A	0.9700	C34—H34A	0.9700
C8—H8B	0.9700	C34—H34B	0.9700
C9—H9A	0.9600	C35—H35A	0.9600
C9—H9B	0.9600	C35—H35B	0.9600
C9—H9C	0.9600	C35—H35C	0.9600
C11—C12	1.416 (12)	C37—C38	1.397 (11)
C11—H11A	0.9700	C37—H37A	0.9700
C11—H11B	0.9700	C37—H37B	0.9700
C12—H12A	0.9600	C38—H38A	0.9600
C12—H12B	0.9600	C38—H38B	0.9600
C12—H12C	0.9600	C38—H38C	0.9600
C13—C14	1.510 (7)	C39—C40	1.500 (7)
C13—H13A	0.9700	C39—H39A	0.9700
C13—H13B	0.9700	C39—H39B	0.9700
C14—H14A	0.9700	C40—H40A	0.9700
C14—H14B	0.9700	C40—H40B	0.9700
C15—C16	1.359 (8)	C41—C42	1.347 (8)
C15—C20	1.510 (8)	C41—C45	1.507 (9)
C16—C17	1.415 (8)	C42—C43	1.401 (8)
C16—C21	1.461 (9)	C42—C47	1.455 (9)
C17—C18	1.366 (8)	C43—C44	1.371 (8)
C17—C24	1.453 (8)	C43—C50	1.466 (9)
C18—C19	1.515 (9)	C44—C46	1.489 (8)
C19—H19A	0.9600	C45—H45A	0.9600
C19—H19B	0.9600	C45—H45B	0.9600
C19—H19C	0.9600	C45—H45C	0.9600
C20—H20A	0.9600	C46—H46A	0.9600
C20—H20B	0.9600	C46—H46B	0.9600
C20—H20C	0.9600	C46—H46C	0.9600
C22—C23	1.443 (12)	C48—C49	1.365 (15)
C22—H22A	0.9700	C48—H48A	0.9700
C22—H22B	0.9700	C48—H48B	0.9700
C23—H23A	0.9600	C49—H49A	0.9600
C23—H23B	0.9600	C49—H49B	0.9600
C23—H23C	0.9600	C49—H49C	0.9600
C25—C26	1.485 (12)	C51—C52	1.407 (11)
C25—H25A	0.9700	C51—H51A	0.9700
C25—H25B	0.9700	C51—H51B	0.9700
C26—H26A	0.9600	C52—H52A	0.9600
C26—H26B	0.9600	C52—H52B	0.9600
C26—H26C	0.9600	C52—H52C	0.9600
			
C10—O2—C11	117.6 (6)	C33—O10—C34	115.4 (5)
C7—O4—C8	118.4 (7)	C36—O12—C37	119.2 (5)
C24—O6—C25	117.4 (5)	C47—O14—C48	115.5 (6)
C21—O8—C22	117.0 (5)	C50—O16—C51	118.1 (6)
C4—N1—C1	114.1 (5)	C27—N3—C30	111.4 (5)
C4—N1—C13	124.2 (5)	C27—N3—C39	124.6 (5)
C1—N1—C13	121.7 (5)	C30—N3—C39	124.0 (5)
C15—N2—C18	107.9 (5)	C44—N4—C41	108.3 (5)
C15—N2—C14	126.8 (5)	C44—N4—C40	125.4 (5)
C18—N2—C14	125.3 (5)	C41—N4—C40	126.4 (5)
N1—C1—C2	106.1 (5)	N3—C27—C28	106.9 (5)
N1—C1—C5	125.6 (6)	N3—C27—C32	123.0 (6)
C2—C1—C5	128.2 (6)	C28—C27—C32	130.0 (6)
C1—C2—C3	106.2 (6)	C27—C28—C29	107.6 (6)
C1—C2—C10	124.2 (6)	C27—C28—C36	124.3 (6)
C3—C2—C10	129.2 (7)	C29—C28—C36	127.4 (7)
C4—C3—C2	108.0 (6)	C30—C29—C28	106.6 (6)
C4—C3—C7	124.0 (6)	C30—C29—C33	124.9 (6)
C2—C3—C7	127.8 (7)	C28—C29—C33	128.3 (6)
N1—C4—C3	105.6 (5)	C29—C30—N3	107.5 (5)
N1—C4—C6	125.5 (6)	C29—C30—C31	128.5 (6)
C3—C4—C6	128.9 (6)	N3—C30—C31	123.8 (6)
C1—C5—H5A	109.5	C30—C31—H31A	109.5
C1—C5—H5B	109.5	C30—C31—H31B	109.5
H5A—C5—H5B	109.5	H31A—C31—H31B	109.5
C1—C5—H5C	109.5	C30—C31—H31C	109.5
H5A—C5—H5C	109.5	H31A—C31—H31C	109.5
H5B—C5—H5C	109.5	H31B—C31—H31C	109.5
C4—C6—H6A	109.5	C27—C32—H32A	109.5
C4—C6—H6B	109.5	C27—C32—H32B	109.5
H6A—C6—H6B	109.5	H32A—C32—H32B	109.5
C4—C6—H6C	109.5	C27—C32—H32C	109.5
H6A—C6—H6C	109.5	H32A—C32—H32C	109.5
H6B—C6—H6C	109.5	H32B—C32—H32C	109.5
O3—C7—O4	121.0 (8)	O9—C33—O10	122.5 (7)
O3—C7—C3	127.7 (8)	O9—C33—C29	124.3 (7)
O4—C7—C3	111.1 (7)	O10—C33—C29	113.0 (6)
C9—C8—O4	109.4 (10)	O10—C34—C35	107.7 (7)
C9—C8—H8A	109.8	O10—C34—H34A	110.2
O4—C8—H8A	109.8	C35—C34—H34A	110.2
C9—C8—H8B	109.8	O10—C34—H34B	110.2
O4—C8—H8B	109.8	C35—C34—H34B	110.2
H8A—C8—H8B	108.3	H34A—C34—H34B	108.5
C8—C9—H9A	109.5	C34—C35—H35A	109.5
C8—C9—H9B	109.5	C34—C35—H35B	109.5
H9A—C9—H9B	109.5	H35A—C35—H35B	109.5
C8—C9—H9C	109.5	C34—C35—H35C	109.5
H9A—C9—H9C	109.5	H35A—C35—H35C	109.5
H9B—C9—H9C	109.5	H35B—C35—H35C	109.5
O1—C10—O2	121.0 (7)	O11—C36—O12	121.9 (6)
O1—C10—C2	123.7 (8)	O11—C36—C28	124.6 (7)
O2—C10—C2	115.2 (6)	O12—C36—C28	113.5 (6)
C12—C11—O2	108.7 (7)	C38—C37—O12	110.6 (7)
C12—C11—H11A	110.0	C38—C37—H37A	109.5
O2—C11—H11A	110.0	O12—C37—H37A	109.5
C12—C11—H11B	110.0	C38—C37—H37B	109.5
O2—C11—H11B	110.0	O12—C37—H37B	109.5
H11A—C11—H11B	108.3	H37A—C37—H37B	108.1
C11—C12—H12A	109.5	C37—C38—H38A	109.5
C11—C12—H12B	109.5	C37—C38—H38B	109.5
H12A—C12—H12B	109.5	H38A—C38—H38B	109.5
C11—C12—H12C	109.5	C37—C38—H38C	109.5
H12A—C12—H12C	109.5	H38A—C38—H38C	109.5
H12B—C12—H12C	109.5	H38B—C38—H38C	109.5
N1—C13—C14	111.0 (4)	N3—C39—C40	111.8 (4)
N1—C13—H13A	109.4	N3—C39—H39A	109.3
C14—C13—H13A	109.4	C40—C39—H39A	109.3
N1—C13—H13B	109.4	N3—C39—H39B	109.3
C14—C13—H13B	109.4	C40—C39—H39B	109.3
H13A—C13—H13B	108.0	H39A—C39—H39B	107.9
N2—C14—C13	112.6 (5)	N4—C40—C39	112.0 (5)
N2—C14—H14A	109.1	N4—C40—H40A	109.2
C13—C14—H14A	109.1	C39—C40—H40A	109.2
N2—C14—H14B	109.1	N4—C40—H40B	109.2
C13—C14—H14B	109.1	C39—C40—H40B	109.2
H14A—C14—H14B	107.8	H40A—C40—H40B	107.9
C16—C15—N2	108.8 (5)	C42—C41—N4	107.9 (5)
C16—C15—C20	131.4 (6)	C42—C41—C45	133.2 (6)
N2—C15—C20	119.6 (6)	N4—C41—C45	118.9 (6)
C15—C16—C17	107.8 (6)	C41—C42—C43	108.5 (5)
C15—C16—C21	122.4 (5)	C41—C42—C47	122.3 (6)
C17—C16—C21	129.6 (5)	C43—C42—C47	128.8 (6)
C18—C17—C16	107.6 (5)	C44—C43—C42	108.2 (5)
C18—C17—C24	123.7 (6)	C44—C43—C50	121.6 (6)
C16—C17—C24	128.0 (6)	C42—C43—C50	129.5 (6)
C17—C18—N2	107.9 (5)	C43—C44—N4	107.2 (5)
C17—C18—C19	132.5 (5)	C43—C44—C46	132.2 (5)
N2—C18—C19	119.5 (6)	N4—C44—C46	120.6 (5)
C18—C19—H19A	109.5	C41—C45—H45A	109.5
C18—C19—H19B	109.5	C41—C45—H45B	109.5
H19A—C19—H19B	109.5	H45A—C45—H45B	109.5
C18—C19—H19C	109.5	C41—C45—H45C	109.5
H19A—C19—H19C	109.5	H45A—C45—H45C	109.5
H19B—C19—H19C	109.5	H45B—C45—H45C	109.5
C15—C20—H20A	109.5	C44—C46—H46A	109.5
C15—C20—H20B	109.5	C44—C46—H46B	109.5
H20A—C20—H20B	109.5	H46A—C46—H46B	109.5
C15—C20—H20C	109.5	C44—C46—H46C	109.5
H20A—C20—H20C	109.5	H46A—C46—H46C	109.5
H20B—C20—H20C	109.5	H46B—C46—H46C	109.5
O7—C21—O8	122.4 (6)	O13—C47—O14	123.9 (7)
O7—C21—C16	124.4 (7)	O13—C47—C42	123.8 (8)
O8—C21—C16	113.2 (5)	O14—C47—C42	112.3 (6)
C23—C22—O8	109.8 (7)	C49—C48—O14	110.0 (8)
C23—C22—H22A	109.7	C49—C48—H48A	109.7
O8—C22—H22A	109.7	O14—C48—H48A	109.7
C23—C22—H22B	109.7	C49—C48—H48B	109.7
O8—C22—H22B	109.7	O14—C48—H48B	109.7
H22A—C22—H22B	108.2	H48A—C48—H48B	108.2
C22—C23—H23A	109.5	C48—C49—H49A	109.5
C22—C23—H23B	109.5	C48—C49—H49B	109.5
H23A—C23—H23B	109.5	H49A—C49—H49B	109.5
C22—C23—H23C	109.5	C48—C49—H49C	109.5
H23A—C23—H23C	109.5	H49A—C49—H49C	109.5
H23B—C23—H23C	109.5	H49B—C49—H49C	109.5
O5—C24—O6	122.9 (6)	O15—C50—O16	121.2 (6)
O5—C24—C17	125.5 (6)	O15—C50—C43	127.1 (6)
O6—C24—C17	111.5 (6)	O16—C50—C43	111.6 (5)
O6—C25—C26	107.2 (7)	C52—C51—O16	111.3 (7)
O6—C25—H25A	110.3	C52—C51—H51A	109.4
C26—C25—H25A	110.3	O16—C51—H51A	109.4
O6—C25—H25B	110.3	C52—C51—H51B	109.4
C26—C25—H25B	110.3	O16—C51—H51B	109.4
H25A—C25—H25B	108.5	H51A—C51—H51B	108.0
C25—C26—H26A	109.5	C51—C52—H52A	109.5
C25—C26—H26B	109.5	C51—C52—H52B	109.5
H26A—C26—H26B	109.5	H52A—C52—H52B	109.5
C25—C26—H26C	109.5	C51—C52—H52C	109.5
H26A—C26—H26C	109.5	H52A—C52—H52C	109.5
H26B—C26—H26C	109.5	H52B—C52—H52C	109.5
			
C4—N1—C1—C2	0.0 (8)	C30—N3—C27—C28	−1.1 (7)
C13—N1—C1—C2	179.5 (6)	C39—N3—C27—C28	−178.5 (6)
C4—N1—C1—C5	−178.7 (7)	C30—N3—C27—C32	177.2 (7)
C13—N1—C1—C5	0.8 (11)	C39—N3—C27—C32	−0.1 (10)
N1—C1—C2—C3	−0.8 (8)	N3—C27—C28—C29	0.7 (7)
C5—C1—C2—C3	177.9 (8)	C32—C27—C28—C29	−177.5 (7)
N1—C1—C2—C10	−174.1 (7)	N3—C27—C28—C36	171.8 (6)
C5—C1—C2—C10	4.6 (13)	C32—C27—C28—C36	−6.4 (11)
C1—C2—C3—C4	1.3 (8)	C27—C28—C29—C30	0.0 (7)
C10—C2—C3—C4	174.1 (8)	C36—C28—C29—C30	−170.8 (7)
C1—C2—C3—C7	175.9 (8)	C27—C28—C29—C33	−176.4 (7)
C10—C2—C3—C7	−11.2 (13)	C36—C28—C29—C33	12.9 (12)
C1—N1—C4—C3	0.8 (8)	C28—C29—C30—N3	−0.6 (7)
C13—N1—C4—C3	−178.7 (6)	C33—C29—C30—N3	175.9 (7)
C1—N1—C4—C6	−176.8 (7)	C28—C29—C30—C31	−176.1 (7)
C13—N1—C4—C6	3.7 (11)	C33—C29—C30—C31	0.4 (11)
C2—C3—C4—N1	−1.3 (7)	C27—N3—C30—C29	1.1 (7)
C7—C3—C4—N1	−176.2 (7)	C39—N3—C30—C29	178.5 (6)
C2—C3—C4—C6	176.2 (8)	C27—N3—C30—C31	176.9 (6)
C7—C3—C4—C6	1.3 (12)	C39—N3—C30—C31	−5.8 (10)
C8—O4—C7—O3	−12.6 (15)	C34—O10—C33—O9	5.2 (11)
C8—O4—C7—C3	172.6 (8)	C34—O10—C33—C29	−180.0 (6)
C4—C3—C7—O3	−31.7 (16)	C30—C29—C33—O9	46.6 (11)
C2—C3—C7—O3	154.5 (11)	C28—C29—C33—O9	−137.6 (8)
C4—C3—C7—O4	142.8 (7)	C30—C29—C33—O10	−128.1 (7)
C2—C3—C7—O4	−31.1 (12)	C28—C29—C33—O10	47.7 (10)
C7—O4—C8—C9	176.5 (10)	C33—O10—C34—C35	−177.7 (7)
C11—O2—C10—O1	−8.6 (13)	C37—O12—C36—O11	2.0 (11)
C11—O2—C10—C2	174.0 (7)	C37—O12—C36—C28	−178.6 (6)
C1—C2—C10—O1	−34.0 (13)	C27—C28—C36—O11	24.6 (11)
C3—C2—C10—O1	154.3 (9)	C29—C28—C36—O11	−166.1 (7)
C1—C2—C10—O2	143.3 (8)	C27—C28—C36—O12	−154.7 (6)
C3—C2—C10—O2	−28.4 (12)	C29—C28—C36—O12	14.5 (10)
C10—O2—C11—C12	178.7 (8)	C36—O12—C37—C38	−176.6 (8)
C4—N1—C13—C14	−98.2 (6)	C27—N3—C39—C40	−83.5 (7)
C1—N1—C13—C14	82.3 (7)	C30—N3—C39—C40	99.5 (6)
C15—N2—C14—C13	−95.1 (7)	C44—N4—C40—C39	−81.7 (7)
C18—N2—C14—C13	84.4 (7)	C41—N4—C40—C39	97.3 (7)
N1—C13—C14—N2	177.8 (6)	N3—C39—C40—N4	−180.0 (6)
C18—N2—C15—C16	0.8 (7)	C44—N4—C41—C42	−0.5 (7)
C14—N2—C15—C16	−179.7 (6)	C40—N4—C41—C42	−179.7 (6)
C18—N2—C15—C20	−175.6 (6)	C44—N4—C41—C45	176.3 (6)
C14—N2—C15—C20	4.0 (10)	C40—N4—C41—C45	−2.9 (10)
N2—C15—C16—C17	−0.9 (7)	N4—C41—C42—C43	1.5 (7)
C20—C15—C16—C17	174.9 (7)	C45—C41—C42—C43	−174.7 (7)
N2—C15—C16—C21	−176.2 (6)	N4—C41—C42—C47	174.4 (6)
C20—C15—C16—C21	−0.4 (11)	C45—C41—C42—C47	−1.7 (12)
C15—C16—C17—C18	0.7 (7)	C41—C42—C43—C44	−1.9 (7)
C21—C16—C17—C18	175.5 (7)	C47—C42—C43—C44	−174.3 (7)
C15—C16—C17—C24	171.3 (7)	C41—C42—C43—C50	−172.0 (7)
C21—C16—C17—C24	−13.8 (12)	C47—C42—C43—C50	15.6 (12)
C16—C17—C18—N2	−0.2 (7)	C42—C43—C44—N4	1.5 (7)
C24—C17—C18—N2	−171.4 (6)	C50—C43—C44—N4	172.6 (6)
C16—C17—C18—C19	176.3 (7)	C42—C43—C44—C46	−177.1 (7)
C24—C17—C18—C19	5.1 (11)	C50—C43—C44—C46	−6.0 (11)
C15—N2—C18—C17	−0.3 (7)	C41—N4—C44—C43	−0.6 (7)
C14—N2—C18—C17	−179.9 (6)	C40—N4—C44—C43	178.5 (6)
C15—N2—C18—C19	−177.4 (6)	C41—N4—C44—C46	178.2 (6)
C14—N2—C18—C19	3.0 (9)	C40—N4—C44—C46	−2.7 (10)
C22—O8—C21—O7	−1.9 (10)	C48—O14—C47—O13	3.0 (12)
C22—O8—C21—C16	179.0 (6)	C48—O14—C47—C42	−175.9 (7)
C15—C16—C21—O7	−54.0 (10)	C41—C42—C47—O13	49.1 (11)
C17—C16—C21—O7	131.8 (8)	C43—C42—C47—O13	−139.4 (8)
C15—C16—C21—O8	125.2 (7)	C41—C42—C47—O14	−131.9 (7)
C17—C16—C21—O8	−49.0 (9)	C43—C42—C47—O14	39.5 (10)
C21—O8—C22—C23	171.4 (8)	C47—O14—C48—C49	163.8 (11)
C25—O6—C24—O5	−5.4 (11)	C51—O16—C50—O15	8.7 (11)
C25—O6—C24—C17	179.3 (6)	C51—O16—C50—C43	−175.2 (6)
C18—C17—C24—O5	−23.1 (11)	C44—C43—C50—O15	28.1 (12)
C16—C17—C24—O5	167.7 (7)	C42—C43—C50—O15	−163.0 (8)
C18—C17—C24—O6	152.1 (6)	C44—C43—C50—O16	−147.8 (6)
C16—C17—C24—O6	−17.2 (10)	C42—C43—C50—O16	21.2 (10)
C24—O6—C25—C26	−173.5 (7)	C50—O16—C51—C52	−173.6 (7)
